# Unfolding biographies—a participatory narrative study on how older adults with multiple sclerosis make sense of and manage their everyday lives

**DOI:** 10.1186/s12877-023-04504-x

**Published:** 2023-12-01

**Authors:** Sofie Olsgaard Bergien, Lasse Skovgaard, Maria Kristiansen

**Affiliations:** 1 The Danish Multiple Sclerosis Society, Poul Bundgaards Vej 1, Valby, 2500 Denmark; 2 Department of Public Health, University of Copenhagen, Oester Farimagsgade 5A, Copenhagen K, Copenhagen, 1014 Denmark

**Keywords:** Aging, Older adults, Multiple sclerosis, Narrative gerontology, Everyday life, Participatory research, User engagement, Biographical aging, Lived experiences

## Abstract

**Background:**

Today, public health research on later life, including the literature on aging with multiple sclerosis, is often centered on aging as a biological phenomenon. By applying a participatory narrative approach, this study conveys how studying biographical aging provides important insights into the elements of aging that people find relevant and meaningful. Based on narratives told by older adults living with multiple sclerosis, we explore how sensemaking unfolds and shapes the management of later life with a chronic and progressive disease.

**Methods:**

Twenty-four older adults (aged 65 years or older) living with multiple sclerosis in Denmark were engaged in taking photographs of their everyday lives and unfold the stories framed in their photographs in subsequent narrative interviews. Interview data were analyzed using a thematic narrative analysis. Aligned with the narrative approach, the findings of the analysis are presented using five cases chosen because they provide insight into the general patterns and themes identified across the narratives of the 24 participants.

**Results:**

Based on their photographs, the participants narrated stories centered around what they perceived as meaningful activities and social identity when aging with a progressive disease. Three themes emerged from the analysis in relation to how participants made sense of and managed aging with multiple sclerosis: 1) a life woven by non-detachable life experiences, 2) envisioning the future and 3) challenging life circumstances.

**Conclusion:**

The findings of the study highlight that aging with multiple sclerosis is not only a biological phenomenon but also something nested in people’s biographies. How people make sense of and manage their everyday lives is shaped by strategies from all parts of their lives—past, present and future. This understanding of later life with multiple sclerosis may enhance the care offered to older adults living with multiple sclerosis if greater emphasis is placed on the exploration of their narratives and the things they find meaningful.

**Supplementary Information:**

The online version contains supplementary material available at 10.1186/s12877-023-04504-x.

## Background

*“*There is not one big cosmic meaning for all; there is only the meaning we each give to our life, an individual meaning, an individual plot, like an individual novel, a book for each person.”—Anaïs Nin [1].

As human beings, we find meaning in our existence through the stories we tell ourselves and others [2]. We “…transform the stuff of our lives into the stories of our lives. And these stories, in turn, become integral to our identity, to our sense of who we are” [3]. This ongoing process can be understood as narrative sensemaking. Based on our life experiences and expectations across past, present and future, we narrate stories to make sense of our situations and find unique ways of adapting to and managing the circumstances in which we are living [3–5].

Narrative sensemaking has been applied across research fields to understand how humans make sense of and manage circumstances such as chronic illness and aging [4–6]. The British sociologist Michael Bury has described how being diagnosed with chronic illness may lead to bodily changes, which in turn can disturb the existing structure of one’s everyday life, forcing people to re-think their sense of self and search for a new destination and purpose in life [7]. Circumstances that can disturb our existing life structures, can be experienced in all life stages but may become more prevalent in older age [3, 8]. Besides illness, the death of a friend or spouse, physical decline or an everyday life with less independence may hinder older adults in their narrative sensemaking, with the consequence that their sense of self diminishes [3]. Some feel that they run out of stories to tell, while others experience a lack of agency in defining and telling their stories [3, 8]. As disturbances in life structures provoke modifications in habits, social roles and identity, this necessitates adaptations in both individuals’ sense of self and their biographies, as new activities or social roles supersede old ones [9, 10]. If such adaptation is not fulfilled, it can potentially affect wellbeing among older adults, as they can experience life as no longer holding any meaning. Regardless of whether older adults find meaning or struggle to make sense of their lives, this influences their behaviors and decisions, which is why the study of narrative gerontology has flourished to understand the complexity of aging [8, 9].

In addition to the individual narratives about who we are, society at large also consists of narratives that influence how we as humans understand the world, each other and ourselves [11]. Such narratives can be seen as master narratives, which shape how phenomena such as aging are understood and acted upon within society [12]. Today, public health research on later life often centers on aging as a biological phenomenon, thereby shaping society’s master narrative on aging to become dominated by stories of physical and mental decline [3, 5, 13]. This tendency is also seen within the literature on aging with the progressive disease multiple sclerosis (MS) [14, 15].

MS is an autoimmune disease affecting 2.8 million people worldwide [16]. With 18,189 people currently living with MS, Denmark has one of the highest rates of prevalence around the globe [16]. For years, MS has been perceived as a “young disease” primarily affecting women aged 20 to 40 [14]. However, due to certain trends in recent decades, including advances in symptom- and disease-modifying treatments, highly specialized rehabilitation and a growing number of people being diagnosed with MS later in life, life expectancy among people with MS is increasing, and the affected population is aging [15, 17]. To date, the majority of research exploring aging with MS has emphasized that the pathology of MS, combined with the pathology process of aging, constitutes an inexpedient course of progression, often leading to physical and cognitive decline [14, 18]. However, it has also been argued that aging with MS must be understood as a complex situation where both biology and contextual circumstances influence aspects of people’s lives [19] and that older adults with MS make sense of and manage their situations individually [20, 21]. In a Danish context, later life with MS has long been neglected, and support offerings have mainly been directed toward younger groups, such as families or young adults. Despite people living with MS in Denmark having the right to free hospital treatment as well as rehabilitation, few—if any—support offerings are designed for or targeted specifically toward older adults living with MS.

To understand later life and potentially support older adults in a way that is meaningful to them, a greater emphasis must be placed on understanding how people narrate their own stories [3]. Contrary to the existing research among older adults living with MS, this study conveys how examining biographical aging provides important insights into elements of aging that people find relevant and meaningful. Based on narratives told by older adults living with MS, we explore how sensemaking unfolds and shapes the management of later life with a chronic and progressive disease. Furthermore, we discuss how this exploration may inform understandings of what aging with MS entails and, importantly, shape supportive interventions that a encompass the individual and biographical embedded needs among the growing population of older adults with MS.

## Methods

### Methodology and study design

This research is part of a larger mixed-method study conducted in Denmark from October 2020 to October 2023. Within this larger context, a pragmatic world view, as described by John W. Creswell and Vicki L. Plano Clark, shaped the research conducted throughout the process [22]. In brief, this means that a practical and applied research philosophy guided our methodological choices, which let our research aim steer our choices of methods rather than an adherence to the belief that “one true” world view exists [22]. As a central part of the research design, we built upon a participatory methodology seeking to engage older adults with MS in the research process. Central to this participatory methodology is the belief that people’s experiences and perspectives are valuable elements of science and that participants should be situated as active subjects engaged in, for example, defining, generating, analyzing and disseminating the research [23, 24]. Within gerontology it has been argued that engaging older adults in research is particularly important to prevent agism and other forms of social exclusion [25].

As a part of the participatory methodology, the present study was conducted in collaboration with an advisory board consisting of eight older adults with MS. Four men and four women aged 65–78 years and diagnosed with MS for six to 26 years participated in activities related to the study design, execution, and analysis. The board members were recruited through The Danish Multiple Sclerosis Society. One had previous experiences with participating in research projects as a “co-researcher.” To engage the advisory board in the research process, we used workshops, face-to-face or phone meetings, online meetings, and email correspondence. Furthermore, as a part of the participatory methodology, we draw on methods that support the involvement of research participants in our data generation [26, 27]. Photovoice [27] and narrative interviews [28] aimed to unfold the lived experiences among older adults with MS in the greatest possible complexity and depth.

### Participants and recruitment

To participate in the study, people had to be 65 years or older and diagnosed with MS. Participants were recruited through The Danish Multiple Sclerosis’s social media and their online and physical magazines, as well as one Danish neurological clinic that displayed a poster for a seven-week period. In total, 24 older adults diagnosed with MS for three to 40 years participated. Participants were 65–81 years old, 14 were female, and 10 were male. Eight of the participants reported having relapsing–remitting MS, eight reported having secondary progressive MS, six reported having primary progressive MS, and two did not know which type of MS they were diagnosed with. Thirteen of the participants used assistive devices for walking, of which, four used a wheelchair. Four participants lived alone. No participants withdrew from the study during the data generation.

### Data generation

#### Photovoice

The data collection was initiated by applying photovoice [27], a visual method introduced by Caroline Wang and Mary Ann Burris. Photovoice enables people to identify, represent and enhance parts of their lives that may not otherwise be available to researchers [27, 29]. Over the years, photovoice has been found to be useful among older adults, whose perspectives are otherwise often neglected, as it creates the opportunity for them to unfold how they experience and cope with illness [30, 31]. Within the present study, photovoice was applied with a twofold and interrelated objective: firstly, to support the involvement of the research participants within the data generation process, and secondly, to gain insight into their experiences of aging with MS as it unfolds within their everyday lives. A photovoice study often consists of smaller or larger group discussions enabling participants to reflect on their photographs, elaborate upon their own and the community’s understandings of the studied phenomenon, and potentially support social change [27]. However, to accommodate the needs of the participants engaged within the present study, the method was modified [30, 32]. During the informed consent process, it became apparent that participants did not consent to sharing their photographs, except with the authors, as they found them too private and sensitive. For the purpose of the present study, the photographs were therefore used to inform individual interviews rather than being shared in a group discussion as had been planned.

When the photovoice was initiated, the participants were asked to photograph elements or situations in their everyday lives that were important and meaningful to them. They were encouraged to take as many photographs as they liked for a minimum of two weeks. In doing so, the participants engaged in co-creating the focus for each of their subsequent individual interviews. Although they were offered cameras to borrow, all participants preferred to use their own devices. Some participants reported needing help from spouses, children or grandchildren to take photographs due to technical challenges or physical limitations. Each participant shared between zero and 62 photographs with the authors, including both photographs taken during the two-week period and pre-existing photographs. One participant did not take photographs but instead wrote a list of topics important to her in her everyday life, as she found it difficult to express herself visually [33]. This participant was not excluded from the study, as the participatory approach encouraged us as researchers to be flexible and responsive to the diverse perspectives and needs of the people included in the research. Her list of everyday life topics served the same purpose as the photographs did for the rest of the participants during the subsequent interviews. In order for the participants to have time to elaborate on their photographs during the interviews, it was decided that subsequent interviews should center around a maximum of 10 photographs. Participants who shared more than 10 photographs with the authors were therefore asked to select which pictures they wanted to include in the interviews. As the main aim of the study was to understand how the participants experienced aging with MS in terms of how they managed their everyday lives, including what they found challenging, the participants were asked to group their photographs into four categories: 1) aging, 2) diseases, 3) support and 4) challenges in everyday life. These categories were chosen both because they could provide some focus in the subsequent interviews [34] and because the advisory board advised that it would help the participants engage in a task which otherwise could be unspecified and difficult to approach. However, the participants were informed that they could bring pictures to the interviews that did not fit into one of the predefined categories, if they considered it important. Furthermore, were all pictures accessible during the interviews for both participants and interviewer (on a computer screen or in print) for them to refer to if it became relevant.

Later in the larger mixed-method study, some of the participants’ photographs were anonymized by being turned into drawings and used in group discussions, between the participants and representatives from the Danish MS Society, revolving around key findings of the overall study and their implications for future practice. In this way, the potentials of photovoice, aiming to promote a critical dialogue through group discussions and reach policy makers, were still achieved [29]. However, neither these stages nor any implications to the study are reported on in this paper.

#### Narrative interviews

Narrative interviews were conducted to enable participants to present their photographs and elaborate on the captured situations. This approach was chosen because it aligns with the participatory approach applied in the study, which aimed to let the participants take the lead in terms of the direction and content of the interviews, but also because it provides a unique insight to people’s lived experiences [26, 28]. The first author, SOB, conducted the interviews approximately one week after participants shared their photographs. The majority of the interviews occurred in the participants’ homes, while one participant preferred that the interview be conducted at The Danish Multiple Sclerosis Society. Each interview comprised four stages, following the narrative approach for conducting interviews [26, 35]. First, the participant was re-introduced to the aim of the research, and the subsequent interview process was explained. In the second step, the participant narrated their stories about aging with MS, with the photographs taken and selected by them used to initiate these stories, with the opening prompt, “Please explain to me what this photograph shows.” If the participant did not elaborate further on the motive captured in a photograph, one of the following questions was asked: “How does that represent how you experience growing older, things that challenge you, living with a disease or things that support you?” When the participant indicated that they had finished their stories (this could occur after each photograph or after the participant had discussed all the photographs), clarifying and exploratory questions were asked as a part of the third stage, if the interviewer found it necessary. After the participant had discussed all their selected pictures, the interview was concluded, and the interviewer explained the next step in the research process and made sure that the interviewee had no further questions. Twenty-three of the interviews were audiotaped and transcribed verbatim. The recoding of one interview was lost, so the field notes taken by SOB during the interview were used for the analysis. This interview was not excluded from the analysis because it adds significantly to the authors’ understanding of aging with MS and therefore informed the overall interpretation of data and engagement with the field. The interviews lasted 51 to 89 min. Since participants did not consent to share their photographs, the results presented in this article are illustrated by the use of interview data.

### Data analysis

**Fig. 1:**
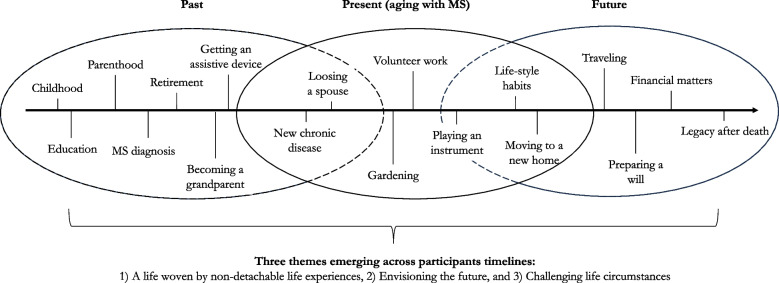
Examples of identified themes as they emerged in relation to the participants’ biographies

We used thematic narrative analysis [26, 28] to explore how older adults make sense of aging with MS, as well as how processes of meaning affect their management of everyday life. Before coding the interviews, the interviews were each read and re-read separately to gain familiarity with the dataset. Aligned with the thematic narrative analysis, we focused on the substances of the stories told, that is, “what” the participants told about aging with MS [36], when the interviews were coded. This were done to understand which moments of personal experiences were meaningful to the participants in relation to aging with MS (see Fig. 1 for examples of themes identified within the participants’ narratives). Furthermore, we focused on “when” these stories occurred to understand how the participants made sense of aging with MS, nested within their biographies (Fig. 1). To do this, we constructed a timeline for each interview, in which we placed the identified themes (the “what” of the participants’ stories) in relation to when they occurred in their biographies. The coding of the interviews was primarily done by the authors SOB and MK, who also each shared their coding and discussed their interpretations of the constructed timelines and the findings emerging from each interview. In face-to-face meetings, the authors and the advisory board discussed their overall understandings of the data and the constructed timelines. Extracts from the participants’ narratives were read aloud three times, followed each time by shared reflections on the question, “What do you think this story is about?” These reflections between the first author and the advisory board enriched the understanding of the participants’ narratives, as the advisory board, embedded in their lived experiences, generated additional insights into how data could be interpretated [37]. Based on the reflexivity emerging in these discussions, the themes and timelines were modified. All 24 timelines were then compared to identify general patterns across interviews in relation to how the participants made sense of aging with MS, as well as how processes of meaning affected their management of everyday life.

Instead of including quotes from all 24 participants, the results section presents data from the following five cases: Tom (71 years old, diagnosed with MS for 40 years), Jennifer (70 years old, diagnosed with MS for 27 years), Caroline (65 years old, diagnosed with MS for 29 years), Summer (66 years old, diagnosed with MS for 8 years) and Rose (81 years old, diagnosed with MS for 32 years). In accordance with earlier practices within narrative research [38, 39], these cases were chosen because they provide insights illustrating general patterns and themes identified across all 24 participants’ narratives and also convey variation in how participants make sense of aging with MS.

## Results

The photographs taken by the participants presented a wide range of objects and situations from their lives, including kitchen gardens, urine bags, swing sets, assistive devices, hiking boots, grandchildren and pets. Based on these photographs, the participants narrated stories centered around what they perceived as meaningful activities and social identity when aging with a progressive disease. As shown in Fig. 1, circumstances such as traveling, becoming a grandparent, building a new home, gardening, playing an instrument or volunteering in the local community were highlighted by the participants when asked to tell stories about how they experience aging with MS. However, the analysis further exhibited that maintaining what they perceived as a meaningful life could be challenged due to the progression of their MS or new diagnoses, leading to existing social roles being changed. In these situations, their stories reflected the need to find a new destination of purpose in life by recreating a sense of meaning, a process embedded in the participants’ biographies. By drawing on e.g. childhood memories, divorces, lessons learned from their work lives, diagnoses or future plans, participants illustrated how aging with MS unfolded in a context consisting of various life circumstances accumulated over a lifetime (Fig. 1). In the majority of narratives, participants positioned themselves as individuals who managed their everyday life based on both their lived experiences and their perceptions of what the future might hold. However, although participants often emphasized their agency through stories of being able to manage their everyday life, their narratives also highlighted experiences where their strategies were insufficient, and they were restrained in relation to acting upon or resolving challenging situations. Based on the three main themes 1) A life woven by non-detachable life experiences, 2) Envisioning the future, and 3) Challenging life circumstances (Fig. 1), the following sections illustrate how sensemaking among participants unfolds in light of their biography as well as how these processes shape the management of their everyday lives.

### A life woven by non-detachable life experiences

Through their narratives, the participants illustrated that a wide range of life circumstances from the past were critical in how they made sense of aging with MS. When the participants experienced that the activities they found meaningful or their social identities were challenged, they drew upon past life experiences to re-make a sense of meaning and an altered definition of purpose in life. As illustrated in Fig. 1, the participants told stories about childhood, living in a violent marriage, traveling alone, dying friends or spouses, learning from a life with MS and work experiences when they narrated how they made sense of potentially challenging situations. Common for these narratives was that participants often used them to illustrate how they—through a lifetime with a wide range of experiences and life circumstances—had developed strategies and experiences that now helped them make sense of and manage everyday life. The following excerpts from the narratives of Tom and Jennifer exemplify how the participants reflected on what life could bring after its current structure was disturbed by MS symptoms or new diagnoses.

Tom, a 71-year-old male living with his wife in a small townhouse, had at the time of the interview been diagnosed with MS for 40 years. The majority of Tom’s narrative on aging with MS centered on finding new meaningful activities to engage in when work was no longer his defining purpose in life or when bodily decline hindered him in activities that were previously part of his social identity. Tom’s narrative about aging with MS began with outlining how he, due to heart failure, had been convinced by his children to retire six years earlier. When Tom was ready to return to work after an operation, his children had asked, “… Daddy, do you really think that [work] is what you should use your energy on the rest of your life?” This question made Tom realize that he needed to start a new chapter in his life and figure out what life could bring when work was no longer the primary source of meaning. Tom explained that he figured out that aging is *“…* an amazing part of life, with so many options to reflect about life and figure out what you would like it to consist of. However, the heavy part is that it is your own responsibility to do so.” This was something that Tom had himself experienced could be difficult, but he also had found ways to manage by drawing on lessons learned from his childhood.

Due to physical limitations caused by MS and heart failure, Tom sold his boat and stopped taking long walks in the woods with his wife. Instead, he found a meaningful community through volunteering in the local municipality, where, together with a group of men, he restored old buildings. During his narrative, Tom showed a photograph of a clock showing the time 8:56 p.m. For him, the clock represented the things he must give up as his mental and physical functions declined—for instance, leaving a social arrangement early because he was too tired to stay. However, Tom also narrated how he makes sense of and manages these situations by drawing on memories of his father and how he was raised as a child:

*He* [my dad] *was a worker and completely worn out, but every time he got worse he started doing something that suited his* [new physical] *level. And that has simply been in my head all along, right? (…) It has been a way of managing difficulties that I bring with me from home. That thing when you get worse, so when I stabilize like that, then you have to, you have to find out, what can you do there, then? What can life bring now? (…) Instead of giving up. Instead of being overwhelmed.*

Through his story about his father being exhausted due to his work as a craftsman, Tom makes sense of the fact that he, due to physical and cognitive decline caused by MS and age, was forced to surrender old interests or adjust how he participates in volunteer work. At the end of his narrative, Tom elaborated that he had not used “formal” supportive interventions such as specialized MS rehabilitation or regular neurologist visits; instead, he has managed his difficulties by using strategies that he had experienced as functioning well for his father.

Like Tom, Jennifer, who was diagnosed with MS more than two decades ago, also used her lived experiences to narrate how she makes sense of aging with MS. Jennifer, at 70 years old, had for many years, since having retired due to her MS, been actively engaged in social work, held positions as a board member in international organizations and traveled the world to make a difference for other people. When Jennifer participated in the study, she had been diagnosed with cancer one year prior—something that dominated her narratives of aging with MS during the interview. As an introduction to her story about aging, Jennifer declared, “I am about to restructure my life—as I no longer only have MS, but now I also have something that is much fiercer.” During her interview, Jennifer showed a map of Europe with crosses representing the cities she had traveled to. When asked about the map, Jennifer explained that it represented both her old life but also how she must find a new purpose in life when she finishes her cancer treatment.

*But I am going to replace this* [traveling around Europe] *because we have decided that I am not going to fly again in my life. It is too dangerous for me. (…). So, this* [the map of Europa] *is about memories, and a way to create a little status, and a way to figure out what my new life should bring. Because now I get my life back. Some people say that I do not have cancer anymore. So, what the hell am I supposed to do with this life?*

In Jennifer’s narrative of aging, the troubling experiences of being diagnosed with MS two decades prior were re-storied into a life event that now aided her sensemaking around living with cancer and finding new meaning in life.

*I am going for a consultation with the surgeon on Friday, where I will be told what the biopsy shows, whether I should have radiation treatment and if I should have more lymph nodes removed. And then I heal on the weekend. (…) So, I have set that week aside to think, because that’s how I’ve done it before. When I got MS, after half a year as a crybaby and lying on the couch, I traveled south three weeks with my son. He was with me the first week, and the last two weeks I spent making a new life career. It’s been damn good and that’s just what I want to do again. But I just need to get those monsters away, right? Where is it, I have to be able to face it.*

Even though Jennifer emphasized that getting cancer had to new circumstances, such as “facing death,” she continued to present herself as a person able to make sense of and act upon her new diagnosis due to the strategies learned from receiving the MS diagnosis decades prior. This theme continuously surfaced in Jennifer’s photographs and narrative. Showing a picture of herself dressed up for her 70^th^ birthday, donning a tiara with golden stars and Danish flags, having only a few tufts of gray hair on her head, Jennifer explained, “There I am, in the chaos of my life. My life, my fear of dying. And it [the photograph] is about holding on: I have done this before… It is a way of showing, that I am trying to take control. You should know, who is inside [the women in the photograph], underneath [what we see on the photograph].”

Both Jennifer’s and Tom’s stories illustrate how many participants connected narratives about earlier experiences to how they manage their current situations and, in that connection, centered their narratives around agency. By drawing on childhood memories and lessons learned from being diagnosed with MS, Tom and Jennifer have managed to adjust to new life circumstances, find new meaningful activities and maintain a sense of self.

### Envisioning the future

In addition to drawing upon experiences from the past, many of the participants’ reflections on what their future might hold were central in their stories about aging with MS and how they have made sense of their lives. Some spoke of planning vacations or emphasized staying physically active and eating healthy in hopes of a “healthy future”; others planned for the future by writing their wills or reflecting on the values they wanted to pass down to their relatives. Often, the participants’ stories pertained to what the future may entail and included reflections on the degree of uncertainty they faced in living with MS. Caroline, a 65-year-old woman who had been diagnosed with MS for more than two decades, initiated her interview with the statement, “I am not a person who worries, not at all. Ehm, and that is really great.” Her subsequent narrative of aging centered around the uncertainty of her future, as well as how she made sense of and adapted through planning to what it might bring.

Caroline lives with her husband in the countryside in a house with a large kitchen garden. Before the interview was initiated, Caroline gave a guided tour in her garden, showing how she was trying to grow vegetables in a new way. The same kitchen garden was also the center of how she initiated her narrative about aging, showing a picture of her garden and her greenhouse.

*The first picture, with just the corner of the greenhouse and the whole kitchen garden… That is simply a major, major part of my life. There could have been a small stool* [in the photograph], *because with becoming older, I have a stool to sit on, which is slightly lower than a dining room chair, such that I can almost fold down over my legs and then reach forward to work. And it is a comfortable position, and then work can begin* [laughs]*. That is how it is to age.*

Finding ways through which she still could maintain her gardening was essential for Caroline in terms of filling her life with meaningful activities despite the physical changes within her body. In addition, Caroline explained that the possibility of not being able to walk one day was difficult for her to make sense of. Caroline’s narrative about living with this fear was sparked by a photograph of herself in a red jacket with a wide smile, sitting on a swing set in her garden built specifically for her to manage this uncertain future, when she potentially would need to find new ways to enjoy nature.

*My husband has made a swing set for me that is five meters high. It’s very difficult to get the swing going. My daughter showed me how to bounce down with your feet back and forth. And down and then forward. Like this* [moves her leg back and forth]*. Okay. I thought, if I suddenly cannot walk, what can I do then? Then I can swing, and why did I think of that? I did it because I was swinging with my grandson in the kindergarten, there was some event down there, sitting and swinging, and when I was done with that, I thought about my oblique abdominal muscles, and I was breathless and everything, and then I thought, if I cannot walk anymore, then I can swing. (…) I have an ambition to swing so high I can see the ocean.*

While the narratives of most participants, such as Caroline, centering on their management of the uncertainty of MS by planning, certain participants emphasized that they did not want to address or think about the future. In these interviews, participants often presented themselves as people who manage the uncertainty by “not worrying.” As one participant explained when asked if he thought about his future, “I’m not the kind of person who worries.” This statement illustrates how some participants showed agency through their narratives by acceptance (e.g., when accepting an unpredictable future).

### Challenging life circumstances

Although the majority of participants illustrated how they make sense of their life circumstances through their lived experiences or by envisioning the future, many also narrated situations they found difficult to act upon. For example, a large share of participants experienced aging and living with MS as implying transitions in their social roles—both private and professional. In their narratives, these participants presented stories on going from being “employed to retired,” “a couple to a widow,” “parent to grandparent” and “caregiver to care receiver.” Summer, a 66-year-old retired nurse who was diagnosed with MS less than a decade ago, took a photograph of the handicap sign in her car. For Summer, the sign represented how she was forced to change her social role due to her age:

*The handicap sign there, for me, represents getting old (…). It’s a different category and a different group than I'm used to being in. I’m used to being the one who can and the one who helps.*

The transition from being a caregiver to becoming a care receiver also unfolded in Rose’s narratives. Rose, who is 81 years old and has been diagnosed with MS for more than three decades, lives with her husband in a home they built together to ensure Rose’s mobility in her wheelchair. Rose still cooks for them at night and makes tea for guests in the kitchen that they have designed to be accessible for Rose in her wheelchair. Central for Rose’s narrative were stories around how she and her husband had handled things on their own, whether searching for proper treatment for her symptoms before they knew she had MS or designing a house where they could live independently. However, Rose was now in a new situation, wherein she expressed her sensemaking being limited by needing care from others, which hindered her doing the things she found meaningful. This story unfolded when Rose showed a photograph of an empty brown armchair in her living room.

*I am dependent on the people of the municipality who come at times that do not fit into my ... Yes, as I would like. I have to be ready in the evening, and I’m not. I never have time to sit down and watch a movie (…). It’s something I miss because we* [Rose and her husband] *could sit and chat together and we could see something, discuss something when we saw or heard something (…). I have to be ready at 10 minutes past 10* [p.m.] *when they come. And we’re typically both night owls, so that’s ... It’s not very nice. (…). We shared the bedroom which we have always done. We had to move my husband out of it, because now I got a lift, because I had to be lifted back and forth from chair to bed and vice versa.*

In Rose’s story, the municipality determines her and her husband’s way of life. Rose’s narrative illustrates how participants experience limitations in their agency when their existing strategies cannot be applied to their new social roles. At one point in the interview, Rose expressed that her life has ceased to be meaningful to her.

*You know what—for my sake, they can say “the party stops here and now” because I have no more.*

Despite this statement of having nothing left to live for, Rose’s narrative amplified a wish to find strategies that could potentially help her to live a more fulfilling life. She explained that new strategies could be identified by listening to peers in this project.

*There could be some who had better ways of dealing with everyday life and some other instruments than the ones I have had that might work better.*

Rose’s narrative illustrates that although some participants felt that their narratives were challenged and struggled to make sense of life within the context of advanced age and disease progression, they maintained the desire to adopt strategies that might help them manage their situations (e.g., changing social roles).

## Discussion

In this participatory narrative study, we explored how aging with MS unfolded in the narratives of older adults living with MS. By engaging 24 older adults in defining the most important aspects to address in a study on later life with MS, the present study gives a unique insight into how aging with MS is experienced in the context of peoples’ biography, consisting of past, present, and imaginable future.

A central finding of the study is how narratives on aging with MS, as told by the participants themselves, stand in striking opposition to the otherwise dominant master narrative of later life with MS. Today, the dominant description of aging with MS centers on biological characteristics, such as cognitive and physical decline, co-morbidities, the risk of dependency and an increased need for support [15, 21, 40–42]—a view on aging also prevalent in society as a whole, coined “the narrative of decline” or “biomedicalized” aging [43, 44]. Within the present study, however, the participants’ narratives illustrate how life domains beyond processes of bodily aging have played a critical role in their experiences and management of aging with MS. This is particularly evident in the life circumstances around which the participants centered their narratives, such as transitions in social roles (e.g., from “a couple to a widower” or “caregiver to care receiver”) or the importance of staying engaged in the everyday life activities that they found meaningful. Such stories about aging can be understood as counterstories—stories that offer resistance to otherwise dominant societal narratives [45].

In the existing literature on aging narratives, counterstories have been highlighted as an important source of societal and individual change [8, 12]. Through unpacking people’s own narratives of aging, circumstances or issues to which we were previously blind can unfold—as people’s stories allow us to get into the “inside of aging” [6, 46]. Further, as people reflect themselves in socially shared stories, they receive an opportunity to find inspiration on how to manage their own lives in new ways, which potentially can increase their wellbeing [13, 45]. Without the counterstories, existing narratives of aging related to bodily decline and dependency will continue to reproduce themselves, with the risk of stigmatizing a group of people and affecting both their mental and physical health [47].

Within the scientific literature on aging with MS, some counterstories have slowly begun to emerge [20, 48–50]. Studies aiming to understand how people adapt to living with MS have described how over time, older adults become “experts” in living with and managing their disease [21, 48–50]. One Canadian study, for example, found that people with MS move through “the path to self-management”—a chronological journey where people transition from “struggling and searching for answers” when they are diagnosed toward self-management later in life [49]. Other studies have, through creative nonfiction stories, provided “positive” narratives about how to “age well” and manage symptoms in ways empowering for the individual [20]. Such counterstories on how MS symptoms can be managed in different ways are important as sources of inspiration for older adults with MS—as well as policy makers, care professionals and patient organizations. Based on the findings of the present study, however, we argue that it is also important to highlight social narratives on aging that are anchored not only in biological changes, strategies for coping with decline, and symptom management but also in how to maintain a sense of self through adapting meaningful activities and social identity. This is particularly relevant because such activities have not only been found to improve wellbeing among older adults with MS but are also something that many experience as difficult to engage in [51, 52]. By sharing stories about how to adapt to changes within social roles and meaningful activities, health care institutions, mass media and patient societies can provide inspiration on how to maintain a sense of self despite aging with MS. We would like to highlight, however—aligned with the findings of earlier studies within narrative gerontology [13]—the importance of social counterstories not becoming overly “positive” and thereby risking to neglect how aging with MS can at times be overwhelming and difficult to make sense of [20] or that having a chronic illness demands ongoing adaptation to one’s everyday life [10]. Instead, the story about aging with MS should be as nuanced, messy and complex as people’s real lives, representing both the agency and the difficulties, constantly evolving and ever changing.

Acknowledging that people make sense of and manage their everyday lives by drawing on their complex and changing biographies implies not only that new social stories about aging with MS must be told but also that the support offered to older adult with MS must be individual and dynamic—considering their sense of self, lived experiences and visions for the future. Based on the findings within the present study, we argue that by understanding where people are coming from (their lived experiences), we can support them in the direction they desire to move (visions for the future) and provide them with support suitable for their specific situations. This argument has also been raised by the literature on patient-centered care—highlighting that delivering care that fits the values, needs and resources of the individual requires an ongoing and iterative process, wherein an understanding of both their *medical* and *personal* history is key [20, 53]. This implies that much greater awareness must be placed on the stories the older adults narrate about their lives—both their present experiences and visions for the future—so that professionals can support them in what they find meaningful. Aligned with previous research in narrative gerontology [3], the present study further highlights that if the support offered to older adults with MS is neglecting the individual’s identity and sense of meaning, it can hinder their wellbeing and purpose in life.

Over the years, multiple strategies have been introduced with the aim of supporting people in sharing their experiences of illness to tailor care and support to their specific situations. One widely used example of these is patient-reported outcomes (PROs), which are designed to support people’s preferences in their treatment and care [54]. Among older adults with MS, PROs have shown potential as a more sensitive measurement of physical function compared to existing measurements [42]. However, instruments such as PROs often only address the immediate context of patients’ lives and have been criticized for exclusively addressing the numbers that represent one’s disease rather than the person living with it [53]. Furthermore, the static nature of PROs (i.e., specific questions in quantitative response categories) may not hold the potential for capturing a more nuanced understanding of an individual’s complex and dynamic life. To further enhance such tools that aim to support older adults with MS in sharing their lived experiences with professionals, integrating open-ended questions that invite conversation could be considered, such as “How do you picture your future?” or “Do you feel you have any experiences that you can draw on in this situation?” Such questions could also be supplemented with visual elements, contributing new ways for people to highlight what is important to them. Among older adults with MS, for instance, it has been found that visual tools such as photography can help put words to parts of their lives otherwise difficult to articulate [33].

Finally, the methodological approach deployed within this study, focusing on doing research *with* the older adults, highlights how a potentially new understanding of a phenomenon can emerge when people are engaged as active (research) partners. Future studies should look into how older adults with MS can best be engaged in developing support offerings targeting them and their special needs.

### Strengths and limitations

This study took its starting point in a participatory methodology with the aims of giving the research participants, 24 older adults with MS, voice and influence throughout the study process and gaining insights into their everyday lives. Through photovoice combined with narrative interviews, this provided a unique opportunity not only to gain insight into how people experience and make sense of aging with MS but also to allow the older adults to decide which stories about aging with MS to tell. Furthermore, working with an advisory board consisting of older adults with MS when analyzing the interview data enabled a richer interpretation. Conducting participatory research should, however, also include reflections related to how the research is shaped by the people engaged in its process, as well as by who is engaged and who is potentially excluded [55]. In the present study, both participants and advisory board members represented a diverse group of older adults living with MS in relation to age, gender, years since diagnosis, education level and place of residence. However, all participants were born in Denmark, and none lived in nursing homes. We therefore note that the narratives explored here do not reflect those of frail older adults (e.g., those living in nursing homes).

As earlier described, the involvement of the advisory board and the research participants is reflected in various decisions made doing the research period. For instance, were adjustments to the method photovoice required to accommodate the older adults’ contributions (e.g., when participants were given the option to participate with written reflections instead of photographs, or when a planned group discussion was replaced with individual narrative interviews). This could be a potential limitation to the quality of the findings, insofar the adjustments were too far from existing methodologies. This can, however, also be considered a strength within the present study, as it allowed for some flexibility, truly letting the older adults influence the research conducted about their lives. In the future, we would encourage researchers across disciplines to share their reflections on how to navigate contributions made by non-academic researchers while remaining true to academic practices.

Finally, we would like to address that only one narrative interview was conducted with each participant. A longitudinal design might have provided more dynamic insight into how narratives develop and change over time [56]. Furthermore, the use of a life history approach could potentially have had highlighted the importance of the participants’ biographies to a greater level than already described in the findings [57]. However, we still believe that the present study design provides a unique insight to how older adults make sense of and manage aging with MS.

## Conclusion

By applying a participatory and narrative approach, the present study contributes to the literature by offering a nuanced understanding of later life with MS, as well as the circumstances that shape how older adults make sense of and manage their life conditions—and ultimately uphold agency. The participants’ narratives further conveyed that aging with MS should be understood as not only a biological phenomenon but also a biographical one, covering social identity and activities considered meaningful. This challenge the existing master narrative of aging with MS and invites us to embrace a more nuanced story of old age with the disease. Furthermore, this implies that not only biological aspects of aging but also social and biographical elements should be considered when designing support offerings for older adults with MS. By applying a participatory approach that combines photovoice and narrative interviews, the study further demonstrates that engaging older adults in qualitative health research can contribute to a more nuanced understanding of the parts of their lives that they find important and meaningful.

### Supplementary Information

**Additional file 1.** Consolidated criteria for reporting qualitative studies (COREQ): 32-item checklist.

## Data Availability

All interview data generated are available upon reasonable request with corresponding author. However, the original photographs cannot be shared as it would compromise the privacy of the participants.

## Abbreviation

MS: Multiple Sclerosis
