# Supplementary material for: Unfolding biographies—a participatory narrative study on how older adults with multiple sclerosis make sense of and manage their everyday lives
Source: BMC Geriatr. 2023 Dec 1;23:794. doi: 10.1186/s12877-023-04504-x (PMC10693063; doi:10.1186/s12877-023-04504-x)
Supplement: Supplementary file 1 — Additional file 1. Consolidated criteria for reporting qualitative studies (COREQ): 32-item checklist. [file 12877_2023_4504_MOESM1_ESM.pdf]

Consolidated criteria for reporting qualitative studies (COREQ): 32-item checklist

No	Item	Guide questions/description	Paragraph and section
**Domain 1: Research team and reflexivity**			
Personal Characteristics			
1.	Interviewer/facilitator	Which author/s conducted the interview or focus group?	Methods – Data generation – Narrative Interviews, paragraph 1
2.	Credentials	What were the researcher's credentials? *E.g. PhD, MD*	SOB: PhD student, MSc Public health<br>LSK: PhD<br>MK: Professor, PhD
3.	Occupation	What was their occupation at the time of the study?	SOB: PhD student<br>LSK: Head of research<br>MK: Professor
4.	Gender	Was the researcher male or female?	SOB: female<br>LSK: male<br>MK: female
5.	Experience and training	What experience or training did the researcher have?	SOB: four years' experience with conducting qualitative research. (Specialized in participatory research, multiple sclerosis, and aging)<br>LSK: 15 years' experience with conducting qualitative research. (Specialized in mixed method research and multiple sclerosis)<br>MK: 15 years' experience with conducting qualitative research. (Specialized in mixed method participatory health section research, narrative approaches, and aging)

**Relationship with participants**			
6.	Relationship established	Was a relationship established prior to study commencement?	MK did not know any of the research participants.<br><br>SOB and LSK are acquainted with one participant prior to the study through their affiliation in the Danish MS Society.
7.	Participant knowledge of the interviewer	What did the participants know about the researcher? e.g. *personal goals, reasons for doing the research*	When the participants signed up for the study, they were informed about the aim of the research.
8.	Interviewer characteristics	What characteristics were reported about the interviewer/facilitator? e.g. *Bias, assumptions, reasons and interests in the research topic*	The participants were informed about the researchers' affiliation as well as SOB's role as a PhD student.
**Domain 2: study design**			
Theoretical framework			
9.	Methodological orientation and Theory	What methodological orientation was stated to underpin the study? *e.g. grounded theory, discourse analysis, ethnography, phenomenology, content analysis*	Background<br>Methods - Methodology and study design, paragraph 1
Participant selection			
10.	Sampling	How were participants selected? *e.g. purposive, convenience, consecutive, snowball*	Methods - Participants and recruitment
11.	Method of approach	How were participants approached? e.*g. face-to-face, telephone, mail, email*	Methods - Participants and recruitment
12.	Sample size	How many participants were in the study?	Methods - Participants and recruitment
13.	Non-participation	How many people refused to participate or dropped out? Reasons?	Methods - Participants and recruitment

Setting			
14.	Setting of data collection	Where was the data collected? e.*g. home, clinic, workplace*	Methods - Data generation – Narrative interviews, paragraph 1
15.	Presence of non-participants	Was anyone else present besides the participants and researchers?	Methods - Data generation – Photovoice, paragraph 2
16.	Description of sample	What are the important characteristics of the sample? *e.g. demographic data, date*	Methods - Participants and recruitment
Data collection			
17.	Interview guide	Were questions, prompts, guides provided by the authors? Was it pilot tested?	Methods - Data generation – Narrative interviews, paragraph 1
18.	Repeat interviews	Were repeat interviews carried out? If yes, how many?	Discussion - Strengths and limitations, paragraph 3
19.	Audio/visual recording	Did the research use audio or visual recording to collect the data?	Methods - Data generation – Photovoice, paragraph 2<br>Methods - Data generation – Narrative interviews, paragraph 1
20.	Field notes	Were field notes made during and/or after the interview or focus group?	Methods - Data generation – Narrative interviews, paragraph 1
21.	Duration	What was the duration of the interviews or focus group?	Methods - Data generation – Narrative interviews, paragraph 1
22.	Data saturation	Was data saturation discussed?	Methods - Participants and recruitment
23.	Transcripts returned	Were transcripts returned to participants for comment and/or correction?	No

**Domain 3: analysis and findingsz**			
Data analysis			
24.	Number of data coders	How many data coders coded the data?	Methods – Data analysis
25.	Description of the coding tree	Did authors provide a description of the coding tree?	Figure 1<br>Methods – Data analysis
26.	Derivation of themes	Were themes identified in advance or derived from the data?	Methods – Data analysis
27.	Software	What software, if applicable, was used to manage the data?	N/A
28.	Participant checking	Did participants provide feedback on the findings?	N/A
Reporting			
29.	Quotations presented	Were participant quotations presented to illustrate the themes / findings? Was each quotation identified? e.*g. participant number*	Results
30.	Data and findings consistent	Was there consistency between the data presented and the findings?	Yes
31.	Clarity of major themes	Were major themes clearly presented in the findings?	Results<br>Figure 1
32.	Clarity of minor themes	Is there a description of diverse cases or discussion of minor themes?	Results

Developed from: Allison Tong, Peter Sainsbury, Jonathan Craig, Consolidated criteria for reporting qualitative research (COREQ): a 32-item checklist for interviews and focus groups, International Journal for Quality in Health Care, Volume 19, Issue 6, December 2007, Pages 349–357, https://doi.org/10.1093/intqhc/mzm042
